# Discerning the Ancestry of European Americans in Genetic Association Studies

**DOI:** 10.1371/journal.pgen.0030236

**Published:** 2008-01-18

**Authors:** Alkes L Price, Johannah Butler, Nick Patterson, Cristian Capelli, Vincenzo L Pascali, Francesca Scarnicci, Andres Ruiz-Linares, Leif Groop, Angelica A Saetta, Penelope Korkolopoulou, Uri Seligsohn, Alicja Waliszewska, Christine Schirmer, Kristin Ardlie, Alexis Ramos, James Nemesh, Lori Arbeitman, David B Goldstein, David Reich, Joel N Hirschhorn

**Affiliations:** 1 Department of Genetics, Harvard Medical School, Boston, Massachusetts, United States of America; 2 Program in Medical and Population Genetics, Broad Institute of Harvard and MIT, Cambridge, Massachusetts, United States of America; 3 Program in Genomics and Divisions of Genetics and Endocrinology, Children's Hospital, Boston, Massachusetts, United States of America; 4 Department of Zoology, University of Oxford, Oxford, United Kingdom; 5 Forensic Genetics Laboratory, Istituto di Medicina Legale, Universita Cattolica del Sacro Cuore, Rome, Italy; 6 Department of Biology, Galton Laboratory, University College London, United Kingdom; 7 Department of Clinical Sciences, Diabetes and Endocrinology, Lund University, University Hospital Malmo, Malmo, Sweden; 8 Department of Pathology, Medical School, National and Kapodistrian University of Athens, Athens, Greece; 9 Amalia Biron Research Institute of Thrombosis and Hemostasis, Sheba Medical Center, Tel Hashomer, Israel; 10 Institute for Genome Sciences and Policy, Center for Population Genomics and Pharmacogenetics, Duke University, Durham, North Carolina, United States of America; University of Chicago, United States of America

## Abstract

European Americans are often treated as a homogeneous group, but in fact form a structured population due to historical immigration of diverse source populations. Discerning the ancestry of European Americans genotyped in association studies is important in order to prevent false-positive or false-negative associations due to population stratification and to identify genetic variants whose contribution to disease risk differs across European ancestries. Here, we investigate empirical patterns of population structure in European Americans, analyzing 4,198 samples from four genome-wide association studies to show that components roughly corresponding to northwest European, southeast European, and Ashkenazi Jewish ancestry are the main sources of European American population structure. Building on this insight, we constructed a panel of 300 validated markers that are highly informative for distinguishing these ancestries. We demonstrate that this panel of markers can be used to correct for stratification in association studies that do not generate dense genotype data.

## Author Summary

Genetic association studies analyze both phenotypes (such as disease status) and genotypes (at sites of DNA variation) of a given set of individuals. The goal of association studies is to identify DNA variants that affect disease risk or other traits of interest. However, association studies can be confounded by differences in ancestry. For example, misleading results can arise if individuals selected as disease cases have different ancestry, on average, than healthy controls. Although geographic ancestry explains only a small fraction of human genetic variation, there exist genetic variants that are much more frequent in populations with particular ancestries, and such variants would falsely appear to be related to disease. In an effort to avoid these spurious results, association studies often restrict their focus to a single continental group. European Americans are one such group that is commonly studied in the United States. Here, we analyze multiple large European American datasets to show that important differences in ancestry exist even within European Americans, and that components roughly corresponding to northwest European, southeast European, and Ashkenazi Jewish ancestry are the major, consistent sources of variation. We provide an approach that is able to account for these ancestry differences in association studies even if only a small number of genes is studied.

## Introduction

European Americans are the most populous single ethnic group in the United States according to U.S. census categories, and are often sampled in genetic association studies. European Americans are usually treated as a single population (as are other groups such as African Americans, Latinos, and East Asians), and the use of labels such as “white” or “Caucasian” can propagate the illusion of genetic homogeneity. However, European Americans in fact form a structured population, due to historical immigration from diverse source populations. This can lead to population stratification—allele frequency differences between cases and controls due to systematic ancestry differences—and to ancestry-specific disease risks [1–5].

Previous studies have carefully analyzed the population structure of Europe [6–8], but here our focus is on European Americans, who constitute a non-random sampling of European ancestry that reflects the historical immigration patterns of the United States. To understand European American population structure as it pertains to association studies, we used dense genotype data from four real genome-wide association studies, analyzing European American population samples from multiple locations in the U.S. We found that in these samples, the most important sources of population structure are (i) the distinction between northwest European and either southeast European or Ashkenazi Jewish ancestry (similar to the main genetic gradient within Europe [6–8]) and (ii) the distinction between southeast European and Ashkenazi Jewish ancestry (which is more readily detectable in our European American data than in previous studies involving Europeans [6–8]). These ancestries can be effectively discerned using dense genotype data, making it possible to correct for population stratification and to identify ancestry-specific risk loci in genome-wide association studies [9].

Although genome-wide association studies that generate dense genotype data are becoming increasingly practical, targeted association studies—such as candidate gene studies or replication studies following up genome-wide scans—will continue to play a major role in human genetics. These studies typically analyze a much smaller number of markers than genome-wide scans, making it far more difficult to infer ancestry in order to correct for stratification and identify ancestry-specific risk loci. To address this, a possible strategy is to infer ancestry by genotyping a small panel of ancestry-informative markers [10], and this is the approach we take in the current paper. Using the insights from analyses of dense genotype data in multiple European American sample sets, we set out to identify markers informative for the ancestries most relevant to European Americans. Important work has already shown that northwest and southeast Europeans can be distinguished using as few as 800–1,200 ancestry-informative markers mined from datasets of 6,000–10,000 markers [7,8]. Here we mine much larger datasets (more markers and more samples) to identify a panel of 300 highly ancestry-informative markers which accurately distinguish not just northwest and southeast European, but also Ashkenazi Jewish ancestry. This panel of markers is likely to be useful in targeted disease studies involving European Americans. In particular, the panel is effective in inferring ancestry and correcting a spurious association in a published example of population stratification in European Americans [1].

## Results

### Analysis of Data from Genome-Wide Association Studies

**Figure 1:**
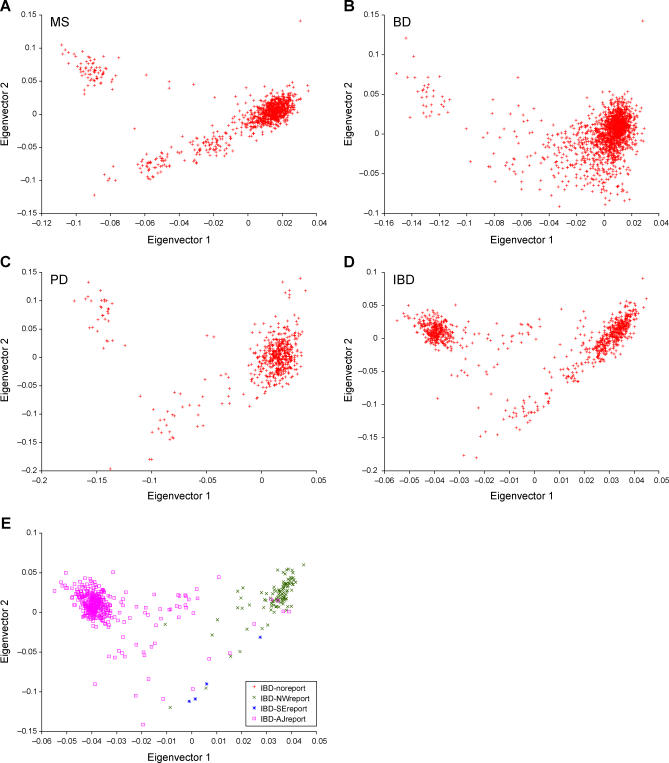
The Top Two Axes of Variation of MS, BD, PD, and IBD Datasets (A) MS dataset, (B) BD dataset, (C) PD dataset, (D) IBD dataset, (E) IBD dataset with samples labeled according to self-reported ancestry (see Methods): northwest European (IBD-NWreport), southeast European (IBD-SEreport) or Ashkenazi Jewish (IBD-AJreport), with individuals having unknown or mixed European ancestry and not self-reporting as Ashkenazi Jewish (IBD-noreport) not displayed.

To investigate whether we could identify consistent patterns of European American population structure, we analyzed four European American datasets involving a total of 4,198 samples. These samples were genotyped on the Affymetrix GeneChip 500K or Illumina HumanHap300 marker sets in the context of genome-wide association studies for multiple sclerosis (MS), bipolar disorder (BD), Parkinson's disease (PD) and inflammatory bowel disease (IBD) (see Methods). For each dataset, we used the EIGENSOFT package to identify principal components describing the most variation in the data [11]. The top two principal components for each dataset are displayed in Figure 1. Strikingly, the results are very similar for each dataset, and are similar to our previous results on a smaller dataset involving the Affymetrix GeneChip 100K marker set [9], suggesting that the main sources of population structure are roughly consistent across European American sample sets.

We were able to characterize the main ancestry components in the IBD dataset, because a subset of these individuals self-reported their ancestry as northwest European, southeast European or Ashkenazi Jewish (see Methods). (We use the term “ancestry” for ease of presentation, but caution that cultural or geographic identifiers do not necessarily correspond to genetic ancestry.) We conclude that the top two principal components of genetic ancestry in the IBD dataset roughly correspond to a continuous cline from northwest to southeast European ancestry and an orthogonal discrete separation between Ashkenazi Jewish and southeast European ancestry (Figure 1E). [We note that the northwest-southeast axis corresponds approximately to the top principal component (x-axis in Figure 1), but this correspondence is not exact, as principal components are mathematically defined to extract the most variance from the data without regards to geographic interpretation. Thus, top principal components will often represent a linear combination of ancestry effects in the data.] Our results are consistent with a previous study in which Ashkenazi Jewish and southeast European samples occupied similar positions on the northwest-southeast axis, although there was insufficient data in that study to separate these two populations [7]. A historical interpretation of this finding is that both Ashkenazi Jewish and southeast European ancestries are derived from migrations/expansions from the Middle East and subsequent admixture with existing European populations [12,13].

**Figure 2:**
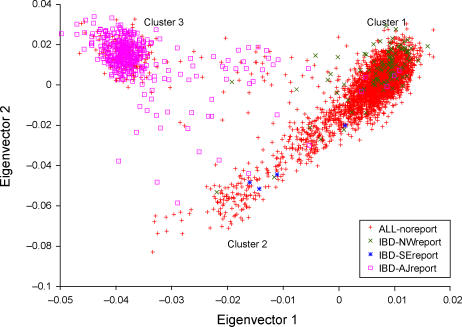
The Top Two Axes of Variation of the Combined Dataset (MS, BD, PD, and IBD) Samples from the IBD dataset are labeled according to self-reported ancestry, as in Figure 1E.

To determine whether the visually similar patterns observed in these four datasets each represent the same underlying components of ancestry, we constructed a combined dataset of MS, BD, PD and IBD samples using markers present in all datasets. The top two principal components of the combined dataset, displayed in Figure 2, are similar to the plots in Figure 1 and show the same rough correspondence to self-reported ancestry labels from the IBD study.

To simplify the assessment of ancestries represented in each dataset, we discretely assigned each sample to cluster 1 (mostly northwest European), cluster 2 (mostly southeast European), or cluster 3 (which contains the great majority of self-reported Ashkenazi Jewish samples) based on proximities to the center of each cluster in Figure 2 (see Methods). We emphasize that this discrete approximation does not fully capture the continuous northwest-southeast cline described by the data, and that we are classifying genetic ancestry rather than cultural or geographic identifiers—for example, not all self-reported Ashkenazi Jewish samples lie in cluster 3. Proportions of individuals assigned to each cluster are listed in Table 1. Results are generally consistent with demographic data indicating that 6% of the U.S. population self-reports Italian ancestry and 2% of the U.S. population self-reports as Ashkenazi Jewish, with higher representation of these groups in urban areas [14,15]. We note that although the self-reported ancestry of samples in the IBD dataset is generally fairly consistent with the cluster assignments, Figure 2 indicates that inferred genetic ancestry is more nuanced and informative than self-reported ancestry with regard to genetic similarity, particularly for individuals who may descend from multiple ancestral populations. By coloring each plot in Figure 1 with cluster assignments inferred from the combined dataset, we verify that the most important ancestry effects in each individual dataset correspond to these clusters (Figure S1).

**Table 1:**
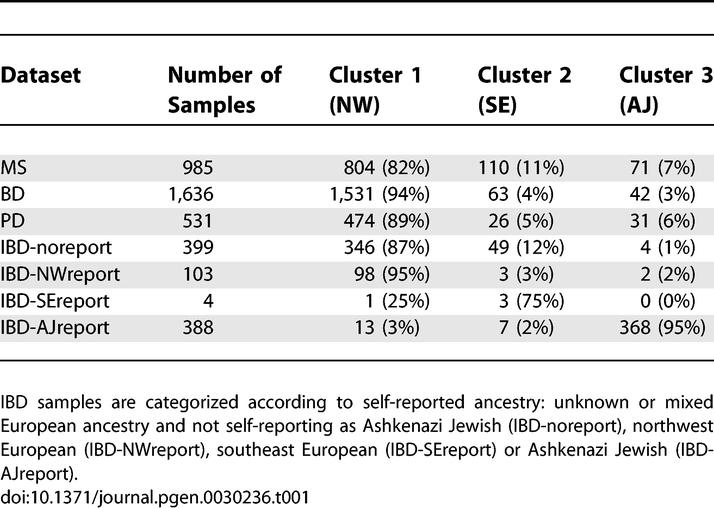
Inferred Ancestry of Individuals in the MS, BD, PD, and IBD Datasets

We computed *F*_ST_ statistics between clusters 1 (mostly NW), 2 (mostly SE) and 3 (mostly AJ), restricting our analysis to individuals unambiguously located in the center of each cluster (Figure 2). We obtained *F*_ST_(1,2) = 0.005, *F*_ST_(2,3) = 0.004 and *F*_ST_(1,3) = 0.009. The additivity of these variances (0.005 + 0.004 = 0.009) would be consistent with the drift distinguishing clusters 1 and 2 having occurred independently of the drift distinguishing clusters 2 and 3, as might be expected under a hypothesis of drift specific to Ashkenazi Jews due to founder effects [13,16]. However, more extensive investigation will be required to draw definitive conclusions about the demographic histories of these populations.

### Impact of European American Population Structure on Genetic Association Studies

To assess the extent to which ancestry differences across sample sets could lead to population stratification in real genetic association studies, we computed association test statistics across the genome, assigning differently ascertained European American sample sets as cases and controls. We first compared the two Affymetrix 500K datasets, treating MS samples as cases and BD samples as controls. (We did not compare the two 300K datasets, which would lead to severe stratification because the IBD dataset was specifically ascertained to include roughly equal numbers of Jewish and non-Jewish samples.) To minimize the effects of assay artifacts [17] on our computations, we applied very stringent data quality filters (see Methods). We computed values of λ, a metric describing genome-wide inflation in association statistics [18], both before or after correcting for stratification using the EIGENSTRAT method [9]. We used the combined dataset to infer population structure, ensuring that the top two eigenvectors correspond to northwest European, southeast European and Ashkenazi Jewish ancestry (Figure 2). Values of λ after correcting along 0, 1, 2 or 10 eigenvectors are listed in Table 2, and demonstrate that the top two eigenvectors correct nearly all of the stratification that can be corrected using 10 eigenvectors, with all of the correction coming from the first eigenvector; the second eigenvector has no effect because the ratio of cluster 2 (SE) to cluster 3 (AJ) samples is the same in the MS and BD datasets (Table 1). Residual stratification beyond the top 10 eigenvectors is likely to be due to extremely subtle assay artifacts that EIGENSTRAT cannot detect – indeed, with less stringent data quality filters (see Methods) the value of λ after correcting for the top 10 eigenvectors increases to 1.090, instead of 1.035.

**Table 2:**
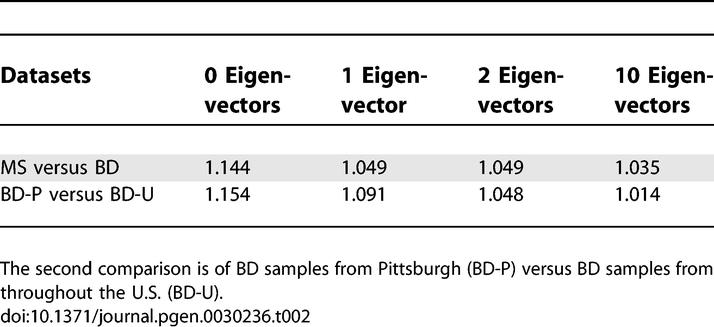
Values of Genome-Wide Inflation Factor (λ) for Two Comparisons of Genome-Wide Datasets, Correcting along 0, 1, 2, or 10 Eigenvectors Using EIGENSTRAT

The BD dataset contains two distinct subsamples (one collected from Pittsburgh and one collected from throughout the U.S.). Thus, we repeated the above experiment using Pittsburgh samples as cases and other U.S. samples as controls and assessed the level of stratification. According to the discrete classification described above, proportions of clusters 1/2/3 ancestry were 91%/8%/2% for Pittsburgh samples vs. 95%/2%/3% for other U.S. samples, thus we would expect differences along the second axis of variation, which distinguishes clusters 2 and 3, to contribute to stratification. Indeed, results in Table 3 show that correcting along the second eigenvector has an important effect in this analysis, and that the top two eigenvectors correct for most of the stratification that can be corrected using 10 eigenvectors.

**Table 3:**
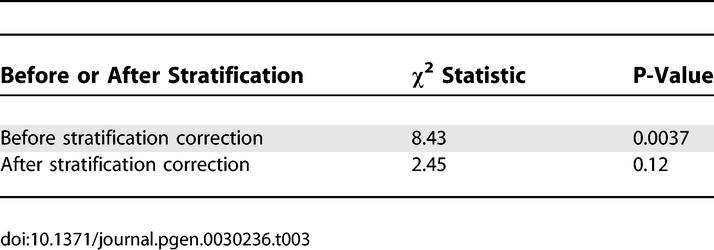
Association Statistics between *LCT* Candidate Marker and Height in 368 European American Samples, before and after Stratification Correction Using Our Panel of 300 Markers

These results suggest that discerning clusters 1, 2 and 3, which roughly correspond to northwest European, southeast European and Ashkenazi Jewish ancestry, is sufficient to correct for most population stratification in genetic association studies in European Americans. However, this does not imply that these ancestries account for most of the population structure throughout Europe, as there are many European populations – such as Russians and other eastern Europeans – that are not heavily represented in the United States [14]. On the contrary, these results, along with the results that follow, are entirely specific to European Americans.

### Validation of a Panel of Ancestry-Informative Markers for European Americans

To develop a small panel of markers sufficient to distinguish clusters 1, 2 and 3 in targeted association studies in European Americans, we used several criteria to select 583 unlinked SNPs as potentially informative markers for within-Europe ancestry (see Methods). These criteria included: (i) Subpopulation differentiation between clusters 1 and 2, as inferred from European American genome-wide data; (ii) Subpopulation differentiation between clusters 2 and 3, as inferred from European American genome-wide data; and (iii) Signals of recent positive selection in samples of European ancestry, which can lead to intra-European variation in allele frequency [19,20]. As we describe below, from these markers we identified a subset of 300 validated markers that effectively discern clusters 1, 2 and 3.

**Figure 3:**
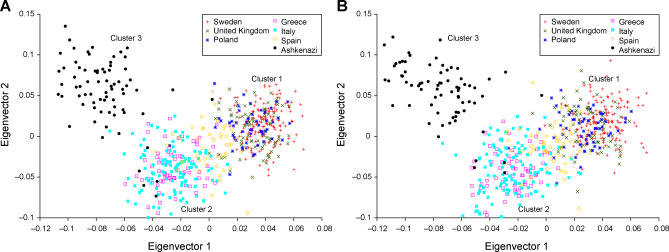
The Top Two Axes of Variation of a Dataset of Diverse European Samples Results are based on (A) 583 markers putatively ancestry-informative markers, and (B) 300 validated markers.

To assess the informativeness of the initial 583 markers for within-Europe ancestry, we genotyped each marker in up to 667 samples from 7 countries: 180 Swedish, 82 UK, 60 Polish, 60 Spanish, 124 Italian, 80 Greek and 81 U.S. Ashkenazi Jewish samples (see Methods). We applied principal components analysis to this dataset using the EIGENSOFT package [11]. Results are displayed in Figure 3A, which clearly separates the same three clusters, roughly corresponding to northwest European, southeast European and Ashkenazi Jewish ancestry, as in our analysis of genome-wide datasets (Figure 2). We note that Spain occupies an intermediate position between northwest and southeast Europe, while Poland lies close to Sweden and UK, supporting a recent suggestion that the northwest-southeast axis could alternatively be interpreted as a north-southeast axis [8].

Defining clusters 1, 2 and 3 based on membership in the underlying populations, we computed *F*_ST_(1,2) and *F*_ST_(2,3) for each marker passing quality control filters, and selected 100 markers with high *F*_ST_(1,2) and 200 markers with high *F*_ST_(2,3) to construct a panel of 300 validated markers (see Methods and Web Resources). We reran principal components analysis on the 667 samples using only these 300 markers, and obtained results similar to before (Figure 3B). The 300 markers have an average *F*_ST_(1,2) of 0.07 for the 100 cluster 1 vs. 2 markers and an average *F*_ST_(2,3) of 0.04 for the 200 cluster 2 vs. 3 markers. These *F*_ST_ values are biased upward since they were computed using the same samples that we used to select the 300 markers from the initial set of 583 markers. However, unbiased computations indicate an average *F*_ST_(1,2) of 0.06 for the 100 cluster 1 vs. 2 markers and average *F*_ST_(2,3) of 0.03 for the 200 cluster 2 vs. 3 markers, indicating that the upward bias is modest (see Methods).

Recent work in theoretical statistics implies that the squared correlation between an axis of variation inferred with a limited number of markers and a true axis of variation (e.g. as inferred using genome-wide data) is approximately equal to *x*/(1+*x*), where *x* equals *F*_ST_ times the number of markers (see Text S1) [21,11]. Thus, correlations will be on the order of 90% for clusters 1 vs. 2 and 90% for clusters 2 vs. 3, corresponding to a clear separation between the clusters (Figure 3B). Because *F*_ST_ is typically above 0.10 for different continental populations, it also follows that these 300 markers (which were not ascertained to be informative for continental ancestry) will be sufficient to easily distinguish different continental populations, as we verified using HapMap [22] samples (Figure S2). Thus, it will also be possible to use these markers to remove genetic outliers of different continental ancestry.

### Correcting for Population Stratification in an Empirical Targeted Association Study

To empirically test how effectively the panel of 300 markers corrects for stratification in real case-control studies, we genotyped the panel in 368 European American samples discordant for height, in which we recently demonstrated stratification [1]. In that study, we observed a strong association (P-value < 10^−6^) in 2,189 samples between height and a candidate marker in the lactase (*LCT*) gene; this association would be statistically significant even after correcting for the hundreds of markers typically genotyped in a targeted association study (or in Bayesian terms, incorporating an appropriate prior probability of association). We concluded based on several lines of evidence that the association was due to stratification—in particular, both *LCT* genotype and height track with northwest versus southeast European ancestry. We focused our attention on a subset of 368 samples and observed that after genotyping 178 additional markers on these samples, stratification could not be detected or corrected using standard methods [1].

**Figure 4:**
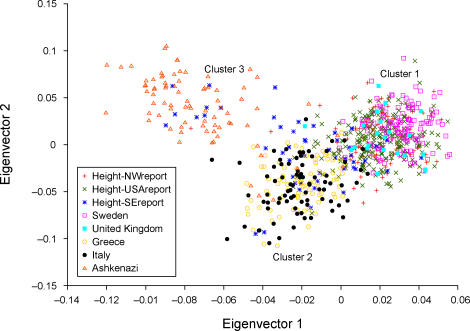
The Top Two Axes of Variation of the Height Samples Together with European Samples Results are based on the 299 markers from our marker panel that are unlinked to the *LCT* locus. Height samples are labeled according to self-reported grandparental origin: northwest European (Height-NWreport), southeast European (Height-SEreport) or four USA-born grandparents (Height-USAreport).

Encouragingly, the panel of 300 markers detects and corrects for stratification in these 368 height samples. We applied the EIGENSTRAT program [9] with default parameters to this dataset, together with ancestral European samples, using the 299 markers unlinked to the candidate *LCT* locus to infer ancestry and correct for stratification (see Methods). We note that it is important to exclude markers linked to the candidate locus when inferring ancestry using a small number of markers, to avoid a loss in power when correcting for stratification [9]. A plot of the top two axes of variation is displayed in Figure 4, with height samples labeled by self-reported grandparental origin (NW Europe, SE Europe, or four USA-born grandparents) as described in the height study [1]. Unsurprisingly, nearly all Height-NWreport samples lie in cluster 1, which corresponds to northwest European ancestry. More interestingly, nearly all Height-USAreport samples also lie in cluster 1; because clusters 2 and 3 do not seem to be represented in the ancestry of USA-born grandparents of living European Americans, the contribution of these clusters to the ancestry of living European Americans may largely descend from foreign-born grandparents, implying relatively recent immigration. Finally, Height-SEreport samples lie in clusters 1, 2 and 3, indicating that self-reported ancestry does not closely track the genetic ancestry of these samples.

We detected stratification between tall and short samples, with the top two axes of variation explaining 5.1% of the variance in height (P-value = 9 × 10^−5^). Furthermore, the top two axes of variation explain 22% of the variance of the candidate *LCT* marker (P-value = 3 × 10^−18^), indicating that the association of the candidate marker to height is affected by stratification. Indeed, the observed association is no longer significant after correcting for stratification (Table 3). The residual trend towards association (P-value = 0.12) could be due to chance, to other axes of variation (besides those corresponding to clusters 1, 2 and 3) which the panel of 300 markers does not capture, or to a very modest true association between *LCT* and height. Our results on genome-wide datasets and on the height dataset suggest that other axes of variation are much less likely to contribute to stratification in European Americans than the main axes we have described. However, the possibility remains that other axes, which are not captured by this panel of 300 markers, could contribute to stratification in some studies.

A recent study reported a successful correction for stratification in the height study using data from the 178 markers that were originally genotyped, using a “stratification score” method [23]. We investigated why the stratification score method succeeded while methods such as STRAT and EIGENSTRAT are unable to correct for stratification using the same data [24,9,1]. The stratification score method computes regression coefficients which describe how genotypes of non-candidate markers predict disease status, uses those regression coefficients to estimate the odds of disease of each sample conditional on genotypes of non-candidate markers, and stratifies the association between candidate marker and disease status using the odds of disease (which ostensibly varies due to ancestry). Importantly, the disease status of each sample is included in the calculation of the regression coefficients that are subsequently used to estimate the odds of disease of that sample. If the number of samples is comparable to the number of markers, then each sample's disease status will substantially influence the set of regression coefficients used to compute the odds of disease of that sample, so that the odds of disease will simply overfit the actual disease status, leading to a large loss in power – even if there is no correlation between disease status and ancestry (see Text S1 and Tables S2 and S3). Thus, we believe that informative marker sets are still needed to allow a fully powered correction for stratification in targeted studies such as the height study.

It is important to point out that the panel of 300 markers provides a better correction for stratification than self-reported ancestry, even for a study in which the ancestry information is more extensive than is typically available. Although the association between the *LCT* candidate marker and height is reduced in the 368 samples when self-reported grandparental origin is taken into account, it is not eliminated (P-value = 0.03). This is a consequence of the fact that grandparental origin explains only 3.2% of the variance in height and 17% of the variance of the candidate marker, both substantially less than is explained by ancestry inferred from the panel of 300 markers. These results provide further evidence that genetically inferred ancestry can provide useful information above and beyond self-reported ancestry [25].

We wondered whether using only the 100 markers chosen to be informative for NW vs. SE ancestry would be sufficient to correct for stratification in the height data. The top axis of variation inferred from these markers explains 19% of the variance of the candidate marker, but only 3.6% of the variance in height. Because this axis captures most of the variation attributable to ancestry at the candidate marker, stratification correction is almost as effective as before (P-value = 0.08). However, this axis is not fully effective in capturing variation attributable to ancestry in height, because it does not separate clusters 2 and 3 – we observed that samples in cluster 2 are strongly biased towards shorter height but samples in cluster 3 show no bias in height in this dataset (data not shown). Thus, although the 100 NW vs. SE markers may be sufficient to correct for stratification in some instances, associations in European American sample sets between other candidate loci and height could be affected by stratification unless the full panel of 300 markers is used. More generally, the complete panel of 300 markers should enable effective correction for stratification in most targeted association studies involving European Americans.

## Discussion

We have analyzed four different genome-wide datasets involving European American samples, and demonstrated that the same two major axes of variation are consistently present in each dataset. The first major axis roughly corresponds to a geographic axis of northwest-southeast European ancestry, with Ashkenazi Jewish samples tending to cluster with southeastern European ancestry; the second major axis largely distinguishes Ashkenazi Jewish ancestry from southeastern European ancestry. We identified and validated a small panel of 300 informative markers that can reliably discern these axes, permitting correction for the major axes of ancestry variation in European Americans even when genome-wide data is not available. We note that while we have corrected for stratification using our EIGENSTRAT method, the panel of markers is not specific to this method, and the STRAT method [24] or other structured association approaches could similarly take advantage of this resource.

Our success in building a panel of markers informative for within-Europe ancestry relied on multiple complementary strategies for ascertaining markers. All strategies were successful in identifying informative markers. We particularly emphasize the success of applying principal components analysis to genome-wide data from European American samples and selecting markers highly differentiated along top axes of variation. This strategy was the source of most of our markers, and will become even more effective as datasets with larger numbers of samples become available, enabling further improvements to the panel and ascertainment of markers to address stratification in other populations.

The panel of 300 markers informative for within-Europe ancestry is practical for genotyping in a small-scale study, and permits correction for population stratification in European Americans at a very small fraction of the cost of a genome-wide scan. We envision three applications:

1. The panel can be used to evaluate study design prior to a genome-wide association study. By randomly choosing a few hundred prospective cases and controls and genotyping them on this panel, one can statistically determine whether or not cases and controls are well matched for ancestry in the overall study. If they are poorly matched, then properly matched cases and controls for the study can be ascertained by genotyping all cases and all controls using this panel (see Text S1).

2. The panel can be genotyped in a targeted association study, such as a candidate gene study or a replication study following up a genome-wide association study, in which variants are targeted in large numbers of samples that have not been densely genotyped. The data from markers in the panel can be used to correct for stratification using methods such as EIGENSTRAT [9], to ensure that observed associations are not spurious. This will also make it possible to search for loci whose disease risk is ancestry-specific [26], without relying on self-reported ancestry.

3. The panel can be used to remove genetic outliers and assess genotyping quality of samples in a targeted association study. Although the panel was not ascertained for evaluating continental ancestry, it is sufficiently informative to identify samples with different continental ancestry (Figure S2). It can also be used to identify duplicate or cryptically related samples.

Though we have focused here on the importance of inferring ancestry in association studies, the panel of markers may prove useful in a broad range of medical and forensic applications.

## Materials and Methods

### Analysis of data from genome-wide association studies.

The MS dataset consists of 1,018 European American parents of individuals with MS that were genotyped at Affymetrix GeneChip 500K markers as part of a trio-design genome-wide scan for multiple sclerosis; most of the individuals (>85%) were sampled from San Francisco. The BD dataset consists of 1,727 European American controls that were genotyped at Affymetrix GeneChip 500K markers as part of a genome-wide scan for bipolar disorder; 1,229 individuals were sampled from throughout the U.S. and 498 were sampled from Pittsburgh. The PD dataset consists of 541 European Americans (270 cases and 271 controls) that were genotyped at Illumina HumanHap300 markers as part of a genome-wide scan for Parkinson's disease [27,28]; individuals were sampled from unspecified locations. The IBD dataset consists of 912 European American controls from the New York Health Project and U.S. Inflammatory Bowel Disease Consortium that were genotyped at Illumina HumanHap300 markers as part of a genome-wide scan for Inflammatory Bowel Disease. [A subset of these samples self-reported their ancestry by indicating one or more of the following: “Scandinavian”, “Northern European”, “Central European”, “Eastern European”, “Southern European”, “East Mediterranean”, or “Ashkenazi Jewish”; we simplified this classification as follows: individuals indicating one or more of “Scandinavian”, “Northern European”, or “Central European” with no other ancestries were reclassified as “IBD-NWreport”, individuals indicating one or more of “Eastern European”, “Southern European”, or “East Mediterranean” with no other ancestries were reclassified as “IBD-SEreport”, individuals indicating “Ashkenazi Jewish” were reclassified as “IBD-AJreport” regardless of other ancestries, and remaining individuals (either unknown or mixed European ancestry and not self-reporting as Ashkenazi Jewish) were reclassified as “IBD-noreport”.] In each of the four datasets, we removed markers with >5% missing genotypes, markers in regions of extended linkage disequilibrium detected as principal components [29], and outlier samples identified by principal components analysis [9]. Analysis of the combined dataset was restricted to ∼50,000 markers present in all datasets after applying these constraints.

### Assignment of samples to three discrete clusters in combined genome-wide dataset**.**

Although the discrete approximation does not fully capture the continuous northwest-southeast cline described by the data, to simplify our analysis we assigned samples to three discrete clusters so as to minimize distances to centers of clusters, defined as (0.01,0.01) for cluster 1, (−0.02,–0.06) for cluster 2 and (−0.04,0.01) for cluster 3 (Figure 2).

### Impact of European American population structure on genetic association studies.

In association analyses involving the MS and BD datasets, we excluded markers that had >1% missing genotypes, or failed Hardy-Weinberg equilibrium (P-value < 0.001), or had a low minor allele frequency (<5%), in either the MS or BD datasets. Roughly 200,000 of the Affymetrix 500K markers remained after imposing these strict constraints. We also repeated our computations with less stringent data quality filters (<5% missing data, instead of <1%).

### Genome-wide datasets used to ascertain ancestry-informative markers.

Genome-wide genotype data used to ascertain markers included the MS, BD, PD and IBD datasets described above, plus three additional European American datasets: a previously described dataset of 488 samples with rheumatoid arthritis (RA) genotyped at Affymetrix GeneChip 100K markers [9], a dataset of 305 unrelated controls from the Framingham Heart Study (FHS) genotyped at Affymetrix GeneChip 100K markers, and a dataset of 297 samples with lung cancer (LC) genotyped at Affymetrix Sty 250K markers. Data from HapMap [22] was also used.

### Ascertainment of putatively ancestry-informative markers.

Markers were ascertained using multiple methods: (i) 185 markers highly differentiated along the top axis of variation in genome-wide datasets. Differentation was defined as the correlation between genotype and coefficient along the top axis of variation, with a correction for sample size. (ii) 300 markers highly differentiated between individuals discretely assigned to cluster 2 (SE) or cluster 3 (AJ) in genome-wide datasets. Differentiation was measured using *F*_ST_, with a correction for sample size. (iii) 112 markers from regions of high *p_excess_* [30] between Europeans and non-Europeans in HapMap [22] data. Regions of high *p_excess_* were identified as windows of consecutive markers with average *p_excess_* values above 0.4, 0.5 or 0.6, comparing allele frequencies in the CEU sample with the pooled YRI+HCB+JPT sample. Within each of the longest such windows, a marker was selected with the highest *F*_ST_ and a higher derived allele frequency in CEU than in the other populations. (iv) 30 markers that were both in the top 1% of the genome for iHH (integrated haplotype homozygosity, a test of recent natural selection) as reported in [19], and also at least 3 standard deviations above the mean in differentiation between European and Asian samples from HapMap. (v) 30 markers that were both in the top 1% of the genome for iHH as reported in [19], and also part of a large stretch of the genome with high iHH (at least two adjacent 100kb regions, as reported in [19]). (vi) 31 markers from our published African American admixture map [30] which in unpublished genotyping results were highly differentiated between European populations from Baltimore, Chicago, Utah, Italy, Norway and Poland, based on the top two axes of variation. (vii) 10 markers highly differentiated between Spanish and European American populations [31]. (viii) 12 markers from the *LCT* gene and *MATP*, *OCA2*, *TYRP1*, *SLC24A5* and *MYO5A* pigmentation genes [30,32,33,7]. Markers which failed primer design or genotyping assay were excluded, yielding a list of 583 putatively ancestry-informative markers.

### Dataset of 667 European samples from seven countries.

The sample collection was assembled on two plates. The first plate included 60 samples from Sweden [34], 60 UK samples from the European Collection of Cell Cultures (ECACC), 60 Polish samples collected by Genomics Collaborative [1], 60 samples from southern Spain and 43 samples from southern Italy [35]. The second plate included 120 additional samples from Sweden, 22 additional UK samples, 81 additional samples from southern Italy, 80 samples from Greece [36] and 81 Ashkenazi Jewish samples from Israel reporting four Jewish grandparents, each born in central or eastern Europe. Genotyping was performed using both the homogeneous MassEXTEND (hME) and iPLEX assays for the Sequenom MassARRAY platform [37]. We genotyped an initial set of 50 markers using the hME assay; for greater efficiency, we used the iPLEX assay to genotype the remaining markers (http://www.sequenom.com/seq_genotyping.html). We designed primers using the Sequenom software, MassARRAY Assay Design. We restricted subsequent analysis to markers that genotyped successfully in >85% of samples and had 1 or fewer discrepancies between replicate samples. We excluded markers with Hardy-Weinberg P-values less than 0.01 in more than one ethnic group.

### Dataset of 368 European American samples.

The samples were collected by Genomics Collaborative/SeraCare, as described previously [1]. Genotyping and quality control were performed as described above.

### Panel of 300 validated markers.

Defining clusters 1, 2 and 3 as described (see Results), we computed *F*_ST_(1,2) and *F*_ST_(2,3) for each marker passing quality control filters. Due to the limited representation of clusters 2 and 3 on the first plate and to minimize differential bias and differences in quality control filters between plates, only the second plate of European samples was used. We first selected 100 markers with the highest *F*_ST_(1,2) and subsequently selected 200 markers with the highest *F*_ST_(2,3), and required that each marker be located at least 1Mb from each previously selected marker. The number of markers from each ascertainment source that were included in the final panel of 300 markers is reported in Table S1.

### Estimates of *F*_ST_ that account for upward bias.

*F*_ST_ values for the panel of 300 markers are biased upward since they were computed using the same samples that we used to select the 300 markers. We computed unbiased estimates of the value of *F*_ST_ for these markers by dividing the samples into four quartiles, with the same distribution of ancestries in each quartile. For each quartile, we selected 300 markers as described above using only samples from the remaining quartiles, then used samples from that quartile to compute unbiased *F*_ST_ values, and averaged the results across quartiles. *F*_ST_ computations were performed using the EIGENSOFT software, which fully accounts for differences in sample size [11].

### Stratification correction of height samples.

We applied the EIGENSTRAT program [9] with default parameters to infer axes of variation of a combined dataset of height samples and the second plate of European samples (see above), using those axes of variation to correct for stratification in the height samples.

### Web resources.

http://genepath.med.harvard.edu/~reich/EUROSNP.htm (panel of 300 markers).

## Supporting Information

Figure S1 The Top Two Axes of Variation of MS, BD, PD, and IBD Datasets Samples are labeled based on cluster assignments inferred from combined dataset (see text).
(1.7 MB PDF)

Figure S2 The Top Two Axes of Variation of HapMap Samples, Using Genotype Data from Our Panel of 300 Markers (96 KB PDF)

Table S1 Number of Markers from Each Ascertainment Source (35 KB DOC)

Table S2 In-Sample versus Out-of-Sample Stratification Score Approaches with Height As Phenotype (31 KB DOC)

Table S3 In-Sample versus Out-of-Sample Stratification Score Approaches with Gender as Phenotype (32 KB DOC)

Text S1 Supplementary Note (43 KB DOC)
