# Supplementary material for: Discerning the Ancestry of European Americans in Genetic Association Studies
Source: PLoS Genet. 2008 Jan 18;4(1):e236. doi: 10.1371/journal.pgen.0030236 (PMC2211542; doi:10.1371/journal.pgen.0030236)

**Price et al.**
**Supp. Figure 1**

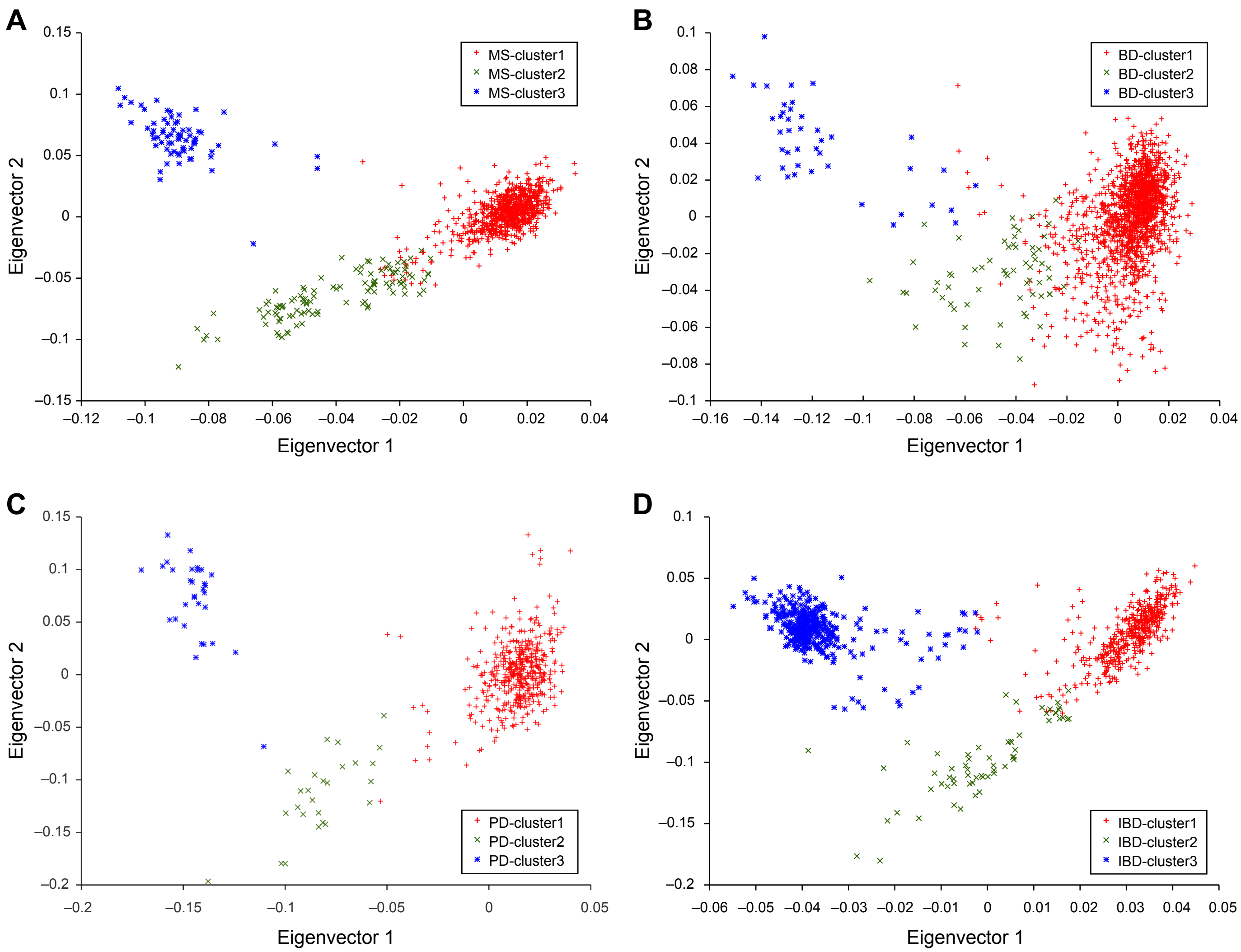

Supplement: Supplementary file 1 [file pgen.0030236.sg001.pdf]
