# Supplementary material for: Discerning the Ancestry of European Americans in Genetic Association Studies
Source: PLoS Genet. 2008 Jan 18;4(1):e236. doi: 10.1371/journal.pgen.0030236 (PMC2211542; doi:10.1371/journal.pgen.0030236)
Supplement: Supplementary file 3 [file pgen.0030236.sd001.doc]

Supplementary Note

Concordance between an inferred axis of variation and a true axis of variation

Let *N* be the number of samples and *M* be the number of markers. According to Theorem 4 of the report of D. Paul, the squared correlation between an inferred axis of variation and a true axis of variation is asymptotically equal to [1-γ/(*l*_1_-1)^2^]/[1+γ/(*l*_1_-1)], where γ = *N*/*M* and *l*_1_ is the leading eigenvalue of the true covariance matrix. From Equation (11) of Patterson et al., we have (*l*_1_-1) = *F*_ST_*N*, thus γ/(*l*_1_-1) = 1/(*F*_ST_*M*). (We caution that Patterson et al. use a different definition of γ than the definition of D. Paul that we follow here.) Assuming that *N* >> 1/*F*_ST_, the squared correlation is approximately equal to 1/[1+γ/(*l*_1_-1)] = *x*/(1+*x*), where *x* = *F*_ST_*M*.

The above result is subject to several assumptions. First, it assumes that γ < (*l*_1_-1)^2^, i.e. that *NM* > 1/*F*_ST_^2^. This corresponds to the BBP phase change of Patterson et al. (the threshold at which population structure is detectable). Because in the current context the aim is not only to detect population structure but also to infer it accurately, this condition will easily be satisfied in applications involving panels of informative markers such as those described in this paper. Second, it assumes that *N* < *M*. More generally, if *N* > *M*, the above formula provides a lower bound on the squared correlation between inferred and true axes of variation. Third, Equation (11) of Patterson et al. assumes two subpopulations of equal size, however the theory can be extended to two subpopulations of size ω*N* and (1-ω)*N*. In this case, the above results hold if we replace *F*_ST_ with 4ω(1-ω)*F*_ST_.

Stratification score method: using disease status to estimate odds of disease

The stratification score method of Epstein et al. computes regression coefficients which describe how genotypes of non-candidate marker predicts the odds of disease, and uses those regression coefficients to estimate the odds of disease of each sample conditional on genotypes of non-candidate markers. The odds of disease is intended to reflect differences in disease risk due to ancestry, as inferred from non-candidate markers.

The genotype data and disease status of sample *j* can either be *included* in the regressions whose coefficients are used to estimate the odds of disease of sample *j* (in-sample approach), or *excluded* from these regressions (out-of-sample approach). Let *N* be the number of samples and *M* be the number of markers. If *N* >> *M*, then the impact of one sample on the regressions will be negligible, so that the distinction between the in-sample and out-of-sample approaches is of little consequence. However, if *N* ~ *M* (or *N* < *M*) and the in-sample approach is used, the combined impact of one sample on the *M* regression coefficients used to estimate the odds of disease of that sample will be substantial, so that the odds of disease will simply overfit the actual disease status – an effect that has nothing to do with ancestry. This will lead to a large loss in power when stratifying the association analysis by the odds of disease. For this reason, we consider the in-sample approach used by Epstein et al. to be inappropriate.

We demonstrate this via a simple reimplementation of the Epstein et al. method which we show yields extremely similar results. In detail, in the height data set using the original 178 markers, for each marker we computed an odds ratio for that marker (the relative risk of being tall per copy of the reference allele) as (*p*_tall_/*p*_short_) / ((1-*p*_tall_)/(1-*p*_short_)) where *p*_tall_ and *p*_short_ are frequencies of the reference allele in tall and short samples. Then, for each sample, we computed the overall odds of disease as the product of relative risks using the number of copies of the reference allele at each marker. Following Epstein et al., we call this the “stratification score” and binned the stratification score into five strata.

We repeated this computation either including (in-sample approach) or excluding (out-of-sample approach) the genotype and height status of sample *j* in the computation of relative risks used to compute the stratification score of sample *j*. Results are displayed in Supp. Table 2. We see that under the in-sample approach, the strata are heavily stratified by height, closely matching the results of Epstein et al., but under the out-of-sample approach, the strata are not significantly stratified by height. Thus, the out-of-sample approach does not effectively stratify to avoid a spurious association in the height study, presumably because the original 178 markers are not sufficiently informative for ancestry.

We performed the same analysis using gender instead of height as a phenotype. We note that gender is essentially a random phenotype, as there is no correlation between gender and ancestry in this sample set. Results are displayed in Supp. Table 3. We again see that under the in-sample approach (but not under the out-of-sample approach), the strata are heavily stratified by phenotype. Stratifying an association analysis via a CMH analysis using these five strata, as recommended by Epstein et al., implies an effective sample size of 242 samples (sum of 4/(1/*N*_male_ + 1/*N*_female_) of each column), implying a loss in power equivalent to a reduction in sample size of 34% – even though there is no underlying ancestry effect with respect to this phenotype. Experiments using randomly generated phenotypes generated very similar results.

Thus, although its correction for stratification in the height study is interesting and intriguing, the in-sample approach used by Epstein et al. will evidently lead to a large loss in power in any data set with 368 samples and 178 markers. Epstein et al. reported that their approach “appears to have negligible effect on power when there is no confounding”, however the number of samples was 10 times larger than the number of markers in all of their simulations, a scenario in which the in-sample approach would be expected to be little different from the theoretically appropriate out-of-sample approach. In summary, informative markers panels are still needed to provide a fully powered correction for stratification in targeted studies such as the height study. (However, the method of Epstein et al. may prove useful in future data sets involving informative marker panels, particularly if *N* >> *M* so that the distinction between the in-sample approach and the out-of-sample approach is unimportant.)

Using the panel of markers to match cases and controls prior to a genome-wide association study

We caution that if one of the ancestry-informative markers is linked to a causal variant, use of these markers to match cases and controls prior to a genome-wide association study could lead to a loss in power. A solution to this problem is as follows: at the time of initial genotyping of all cases and controls at the ancestry-informative markers, each of these markers is tested for disease association (correcting for possible stratification using all other ancestry-informative markers). Any marker showing a suggestive association (significance beyond some nominal threshold) is then removed from the set of ancestry-informative markers used for matching. A subset of cases and controls is then matched using the remaining ancestry-informative markers, and genotyped in the genome-wide association study.
