# Supplementary material for: Discerning the Ancestry of European Americans in Genetic Association Studies
Source: PLoS Genet. 2008 Jan 18;4(1):e236. doi: 10.1371/journal.pgen.0030236 (PMC2211542; doi:10.1371/journal.pgen.0030236)
Supplement: Supplementary file 4 [file pgen.0030236.st001.doc]

Supplementary Table 1: Number of markers from each ascertainment source that were included in the final panel of 300 markers. Ascertainment sources are described in detail in the Methods section.

| Ascertainment source | #markers ascertained | #markers in final panel |
| --- | --- | --- |
| (i) GWAS data, top axis | 185 | 88 |
| (ii) GWAS data, cluster 2 vs. 3 | 300 | 163 |
| (iii) *p_excess_* | 112 | 22 |
| (iv) iHH & EUR vs. ASI | 30 | 9 |
| (v) iHH & adjacent iHH | 30 | 4 |
| (vi) AA admixture map | 31 | 8 |
| (vii) SPAN vs. EUR | 10 | 4 |
| (viii) LCT/pigmentation genes | 12 | 2 |
