# Supplementary material for: Discerning the Ancestry of European Americans in Genetic Association Studies
Source: PLoS Genet. 2008 Jan 18;4(1):e236. doi: 10.1371/journal.pgen.0030236 (PMC2211542; doi:10.1371/journal.pgen.0030236)
Supplement: Supplementary file 5 [file pgen.0030236.st002.doc]

Supplementary Table 2: Number of tall/short samples in each of the five stratification score strata inferred using the height data with height as phenotype, using either the in-sample or out-of-sample approach (see text).

|  | Stratum 1 | Stratum 2 | Stratum 3 | Stratum 4 | Stratum 5 |
| --- | --- | --- | --- | --- | --- |
| In-sample | 68/6 | 53/21 | 41/32 | 18/56 | 12/61 |
| Out-of-sample | 43/31 | 39/35 | 35/38 | 34/40 | 41/32 |
