# Supplementary material for: Discerning the Ancestry of European Americans in Genetic Association Studies
Source: PLoS Genet. 2008 Jan 18;4(1):e236. doi: 10.1371/journal.pgen.0030236 (PMC2211542; doi:10.1371/journal.pgen.0030236)
Supplement: Supplementary file 6 [file pgen.0030236.st003.doc]

Supplementary Table 2: Number of male/female samples in each of the five stratification

score strata inferring using the height data with gender as phenotype, using either the in-sample or out-of-sample approach (see text).

|  | Stratum 1 | Stratum 2 | Stratum 3 | Stratum 4 | Stratum 5 |
| --- | --- | --- | --- | --- | --- |
| In-sample | 67/7 | 60/14 | 37/36 | 23/51 | 10/63 |
| Out-of-sample | 39/35 | 39/35 | 42/31 | 38/36 | 39/34 |
